# A Case of Poisoning by Prussic Acid

**Published:** 1872-10-01

**Authors:** H. Wardner

**Affiliations:** Cairo, Ill.


### A CASE OF POISONING BY PRUSSIC ACID.

BY H. WARDNER, M.D., CAIRO, ILL.

On the 25th of May, 1865, I was called to see a Mrs. G——, who was said to have been found alone in her house, "dying in a fit." I first saw her about 8:30, p. m. She was lying on her back upon a pallet on the floor, breathing with difficulty and great labor, and yet with a good deal of regularity; the effort at expiration was more difficult and labored than that of inspiration. There was some frothing at the mouth; the face was flushed, and the veins about the neck and head greatly distended with blood; the eyes were wide open and glaring, the eyelids fixed, the eyeballs were seen to have an oscillating motion, the pupils were about the natural size; the extremities were cold; the pulse was very feeble and much slower than natural; there was entire loss of sensation.

Poison was at once suspected, but no trace of anything indicating its use could be found about the premises. She was previously a healthy woman, and had never been subject to apoplectic, cataleptic, or epileptic symptoms. There was no smell of any drug in the breath of the patient. A stomach pump was obtained, but found to be unserviceable. The patient being too far gone to qe affected by emetics, and not being able to ascertain the cause of her condition, it was determined to treat the case as the symptoms seemed to indicate.

The treatment consisted first of pouring ice cold water from a height of two or three feet upon her head during fifteen or twenty minutes. As this was being done, her breathing gradually became easier. Second, The median vein of the left arm was freely opened, and the blood flowed in a small slow stream, to the amount of four ounces. Third, The head was then surrounded with ice, and hot mustard applications made to the feet and legs, while she inhaled the vapor of ammonia. During the flowing of the blood from the arm some convulsions occurred, which lasted only five minutes. The breathing then became natural. After an interval of ten minutes there was a recurrence of convulsions, but less severe than the first, after which no more occurred. With my mouth close to her ear I called her name in a loud tone of voice, when she gave evidence of hearing and of returning sensation. About half past ten o'clock she could respond to the calling of her name, when I left her.

The next morning when I called to see her, she was able to come to the door, but seemed averse to conversation. I prescribed a cathartic.

Three days afterwards I found her suffering severely with hemorrhoids, to which she had been subject. That evening I was notified to see her at a certain hour, when I would be put in possession of some important facts in the case. At the appointed time, after due cautions, admonitions, and threats, about the secret being kept, she went on to state:

"I have attempted to take my own life and you have prevented me. I have been in much domestic trouble. I am a forsaken wife; I have nothing to live for and no desire to live. I took prussic acid; I got it at a certain drug store, telling them I wanted it to use in preserving the skins of birds for stuffing. The druggist told me one drop would be sure death; it was an ounce vial full; it had a glass stopper which was sealed in the vial. I sent my sister out on an errand, thinking she would be gone until I was dead. I then took an iron wedge, a stout cord, and the vial of poison, and went to the privy; I tied one end of the cord around the wedge and the other around the neck of the vial; I then drew the stopper and threw it into the vault, then lowered the iron wedge through the hole in the seat. I then swallowed the contents of the vial and dropped it where it was carried with the wedge to the bottom of the vault, so that nothing remained to indicate what I had done. I expected to drop down dead at once; I sat down on the seat a moment, and as I did not feel the poison, got up and came into the house. As I entered the back door I staggered, but succeeded in reaching the parlor, when I felt my head go round. That is all I remember until the next day. I took all there was in the vial."

This patient was not long in regaining her usual health, and husband; but on account of subsequent estrangement, she was more successful in a second attempt "to shuffle off this mortal coil," which terminated her life and sorrows, about a year after the first trial.

Supposing the acid used to have been the U. S. preparation, it would have contained two per cent. of the anhydrous acid, an amount equal to the destruction of more than one human life. The case was one of considerable interest to me on account of the large quantity of the poison taken, and the result of the treatment (which was not exactly according to the books), as well as the *sang-froid* with which she went about the business. As cases of poisoning by this agent are not very frequent, I send the above statement, thinking it may be of interest to some of your many readers.

